# Evaluating stereotactic accuracy with patient‐specific MRI distortion corrections for frame‐based radiosurgery

**DOI:** 10.1002/acm2.14472

**Published:** 2024-07-23

**Authors:** Cory Knill, Robert Halford, Raminder Sandhu, Brian Loughery, Sharjil Shamim, Fred Junn, Kuei Lee, Muayad Almahariq, Zachary Seymour

**Affiliations:** ^1^ Department of Radiation Oncology Corewell Health William Beaumont University Hospital Royal Oak Michigan USA; ^2^ William Beaumont School of Medicine Oakland University Rochester Michigan USA

**Keywords:** distortions, MRI, SRS

## Abstract

**Purpose:**

This study examines how MRI distortions affect frame‐based SRS treatments and assesses the need for clinical distortion corrections.

**Methods:**

The study included 18 patients with 80 total brain targets treated using frame‐based radiosurgery. Distortion within patients' MRIs were corrected using Cranial Distortion Correction (CDC) software, which utilizes the patient's CT to alter planning MRIs to reduce inherent intra‐cranial distortion. Distortion was evaluated by comparing the original planning target volumes (PTV_ORIG_) to targets contoured on corrected MRIs (PTV_CORR_). To provide an internal control, targets were also re‐contoured on uncorrected (PTV_RECON_) MRIs. Additional analysis was done to assess if 1 mm expansions to PTV_ORIG_ targets would compensate for patient‐specific distortions. Changes in target volumes, DICE and JACCARD similarity coefficients, minimum PTV dose (*D*_min_), dose to 95% of the PTV (D95%), and normal tissue receiving 12 Gy (*V*_12Gy_), 10 Gy (*V*_10Gy_), and 5 Gy (*V*_5Gy_) were calculated and evaluated. Student's *t*‐tests were used to determine if changes in PTV_CORR_ were significantly different than intra‐contouring variability quantified by PTV_RECON_.

**Results:**

PTV_RECON_ and PTV_CORR_ relative changes in volume were 6.19% ± 10.95% and 1.48% ± 32.92%. PTV_RECON_ and PTV_CORR_ similarity coefficients were 0.90 ± 0.08 and 0.73 ± 0.16 for DICE and 0.82 ± 0.12 and 0.60 ± 0.18 for JACCARD. PTV_RECON_ and PTV_CORR_ changes in *D*_min_ were –0.88% ± 8.77% and −12.9 ± 17.3%. PTV_RECON_ and PTV_CORR_ changes in D95% were −0.34% ± 5.89 and −8.68% ± 13.21%. The 1 mm expanded PTV_ORIG_ targets did not entirely cover 14 of the 80 PTV_CORR_ targets. Normal tissue changes (*V*_12Gy_, *V*_10Gy_, *V*_5Gy_) calculated with PTV_RECON_ were (−0.09% ± 7.39%, −0.38% ± 5.67%, −0.08% ± 2.04%) and PTV_CORR_ were (−2.14% ± 7.34%, −1.42% ± 5.45%, −0.61% ± 1.93%). Except for *V*_10Gy_, all PTV_CORR_ changes were significantly different (*p* < 0.05) than PTV_RECON_.

**Conclusion:**

MRIs used for SRS target delineation exhibit notable geometric distortions that may compromise optimal dosimetric accuracy. A uniform 1 mm expansion may result in geometric misses; however, the CDC algorithm provides a feasible solution for rectifying distortions, thereby enhancing treatment precision.

## 1 INTRODUCTION

Stereotactic radiosurgery (SRS) employs magnetic resonance imaging (MRI) for target delineation in the treatment of brain metastases with or without computed tomography (CT).^1^ While MRIs offer excellent soft tissue contrast with the capacity to evaluate imaging in multiple planes, they are also inherently susceptible to geometric distortion that may affect spatial accuracy.^2^, ^3^, ^4^ This distortion arises in part due to inhomogeneities of the magnetic field and magnetic susceptibility of the object being imaged. The magnetic field's characterization can be incorrect, leading to unknown perturbations that can shift the targets in the MRI.^5^ Some common examples include the “potato‐chip” and “bowtie” effects. Planning on an MRI without CT for dose calculation could potentially exacerbate dosimetric treatment inaccuracies.^6^ Despite these challenges, MRIs remain crucial for detecting and contouring soft tissue cranial SRS targets and distortion‐induced errors persist in routine clinical practice.

Alzahrani et al. conducted an audit of 11 MR scanners used for target contouring in radiotherapy in the UK and found that the mean geometric distortions for head scanning protocols were <2 mm, but the maximum distortion was observed to be >10 mm.^7^ Previous studies using polymer gel‐filled phantoms have measured the magnitudes of scanner‐specific MRI distortions to be <1 mm.^8^ Distortions due to the magnetic susceptibility of the head in a 3T MRI have been generally found to be <1 mm, but up to 4 mm in some cases.^9^ The cumulative effect of these distortions could lead to inaccurate target contouring and result in a geometric treatment miss, albeit in a minority of cases, but with clear clinical implications when they arise.^10^

Due to the critical role of geometric accuracy in MRIs, significant efforts have been made to reduce the effects of spatial distortions. One approach is to acquire a second scan with the phase‐encoded gradient polarity reversed and averaging the scans to reduce distortion caused by non‐linear gradients and the utilization of 3D acquisition sequences.^11^ Although efforts have been made to minimize the inherent distortion in MRI scanners, the residual inherent distortion combined with the patient‐specific distortion can be of the same order of magnitude as the target sizes treated in cranial SRS and are larger than typical alignment errors.^12^, ^13^ To address distortion and other uncertainties in SRS treatment paradigms, a uniform margin can be added to target volumes; however, this would not account for the fact that distortion tends to be non‐uniform and increase in magnitude with distance from the image center.^14^ Distortions may have more significant dosimetric effects on small targets, where geometric shifts place a higher percentage of the target outside the treatment volume compared to larger targets.^15^ Therefore, additional margins to reduce the risk of geometric misses in the absence of patient‐specific distortion corrections may need to be determined based on target size and location.

Given the non‐uniform nature of distortion within the image and the variable effects of distortion on different target sizes, patient‐specific distortion corrections are an attractive option for improving geometric accuracy as this would account for distortion based on each target on any given MRI. One solution would be to allow software to correct the distortion inherent to the planning MRI by adapting it to a planning CT. The Elements Cranial Distortion Correction (CDC) software (Brainlab AG, Munich, Germany) used in this study assumes the anatomy within the CT is free from geometric distortion and adjusts the MRI anatomy to create a distortion‐corrected MRI that corresponds to the CT. Previous research has demonstrated that this algorithm can reduce the maximum distortion in an MRI to approximately 1.0 mm, with 96% of the voxels having distortion < 0.5 mm.^16^ In this study, the CDC algorithm was utilized to investigate the dosimetric impact of residual MRI distortions on frame‐based treatment plans for brain metastases that used a clinical stereotactic‐commissioned MRI for target delineation.

## 2 METHODS

To investigate the impact of MRI distortion on SRS plans, the CDC software was retrospectively applied to correct clinical planning MRs used for target delineation. The corrected MRs were used to re‐contour the targets, and the differences in dose between the original planning targets and the corrected targets were quantified. Additionally, the MRI targets were re‐contoured on the original MRs and compared with the original planning targets to evaluate the extent to which changes in corrected versus original targets were due to intra‐contouring variability.

### 2.1 Initial patient treatments

The study included 18 patients with a total of 80 previously treated brain metastases using frame‐based radiosurgery. Target sizes ranged from 0.01 to 12.38 cc and were located throughout different regions of the brain. All treatments consisted of a single fraction with doses ranging from 15 to 20 Gy. Prior to treatment, all patients underwent an MRI and CT scan for planning purposes. MRIs were acquired on a Siemens Symphony TIM 1.5T MR scanner using a gadolinium‐enhanced T1 fast low angle shot (FLASH) scanning protocol with 2D distortion correction applied. The FLASH sequence served as the institution's standard clinical MRI protocol for brain metastases, chosen for its historical usage and physician's familiarity with delineating targets using this MRI sequence. The axial MR images were obtained using the following scanning parameters: a field‐of‐view of 256 mm, matrix size of 256 × 256, TR of 1.1 ms, TE of 3.5 ms, bandwidth of 130 Hz/pixel, flip angle of 18.14, and a slice thickness of 1 mm. The MR scanner was commissioned for cranial SRS within the hospital and followed the AAPM and ACR recommendations for rigorous quality assurance.^17^, ^18^ CT images were acquired immediately after the MRI using the Radiation Oncology department's Philips Big Bore CT scanner or Radiology's Siemens Somatom Sensation 16 CT with a head scanning protocol. The CT scanning parameters included a field‐of‐view of 278 mm, axial pixel size of 1 mm, slice thickness of 1 mm, a kVp of 120 kV, and a focal spot size of 1.2 mm. The CT quality assurance of both machines followed AAPM TG‐66 guidelines.

After acquiring CT and MR images, the patient was moved to a holding area, and the images were imported into the Leksell Gamma Plan (LGP) software (Elekta AB, Stockholm, Sweden) for contouring and treatment design. CT and MRI images were co‐registered, and targets were contoured using the gadolinium‐enhanced regions of the MRI without any additional margin. The accuracy of the co‐registration and target contours was confirmed by the neurosurgeon, radiation oncologist, and medical physicist before initiating treatment. Dose prescriptions were based on target size. All patients were treated using a Gamma Knife Icon, which is composed of a hemisphere of 192 radioactive Cobalt‐60 sources that are precisely focused on a specific location within the unit, known as isocenter with a frame‐based treatment.^19^ The TMR 10 algorithm was utilized to calculate dose, with a voxel size of 0.5 mm on the planning CT. Sector blocking, various collimators, and inverse and forward planning were all utilized based on the individual target to create an optimal dose plan in terms of dose, gradient, and conformity.

The planner's goal was to achieve 100% target coverage with the prescription isodose line, which was typically set at 50% of maximum dose, while minimizing radiation dose to the surrounding normal brain tissue.

### 2.2 Distortion investigation

**FIGURE 1:**
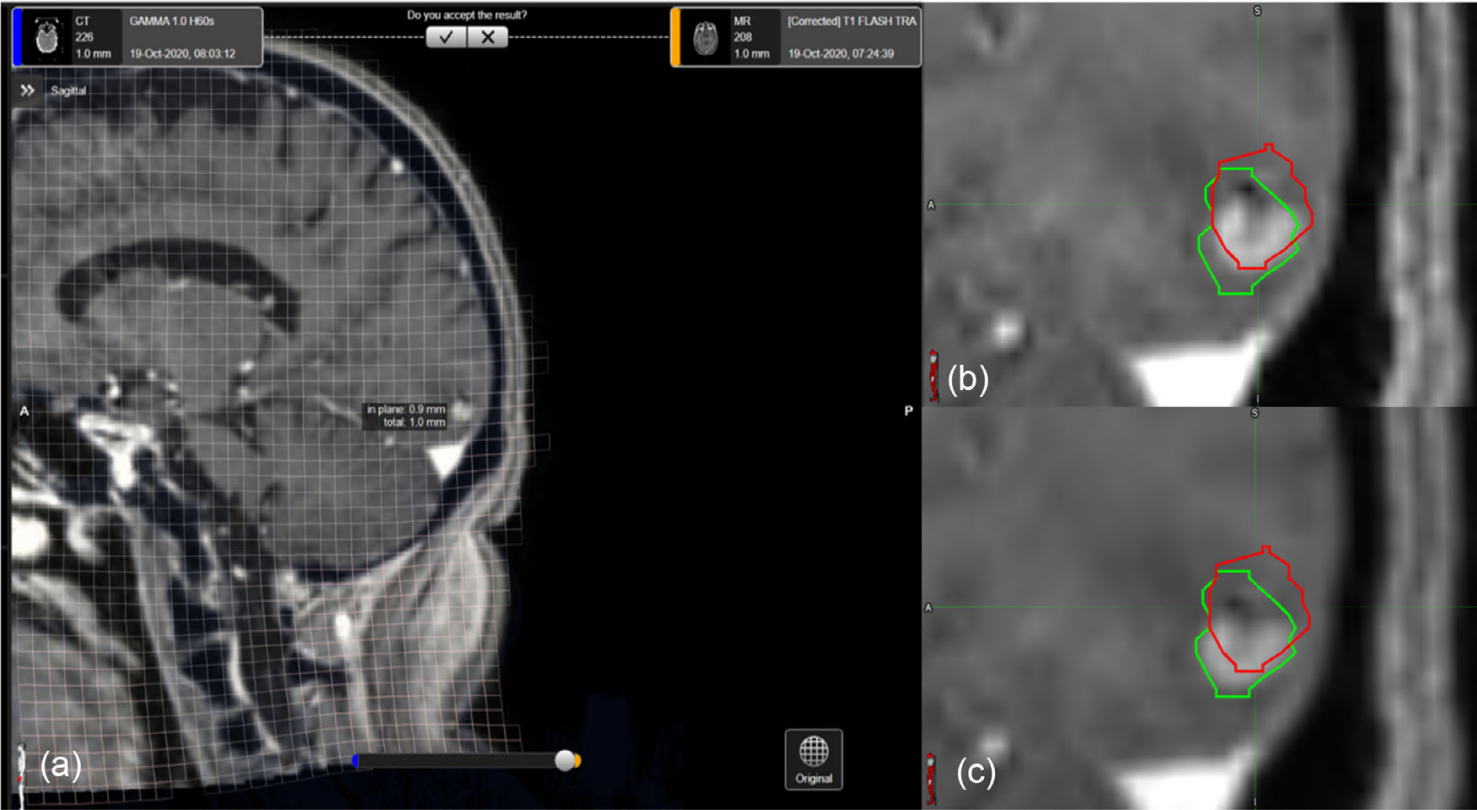
Cranial distortion correction of an MRI using the CDC algorithm. (a) shows the CDC interface displaying the distortion grid. (b) shows the original (red) and corrected (green) contours overlaid on the uncorrected MRI. (c) displays the final corrected MRI.

To initiate the investigation of MR distortion, MR and CT planning images, MR‐CT registration, target structures, and dose DICOM files were exported from LGP to the MIM software (MIM Software Inc., Beachwood, OH). MIM was used for all structure and dose analysis in the study. Once all the LGP data were imported into MIM, MR and CT images were exported into the CDC software. An example CDC correction, along with the PTV_ORIG_ and PTV_CORR_ on the original and corrected MRIs, is presented in Figure 1. Co‐registration of MR and CT images was performed, and the CDC algorithm was applied, resulting in a corrected MRI that was co‐registered to the CT. The CDC algorithm employs a 5‐step process to address distortion within the MRI. First, the MRI is co‐registered to the CT. Next, the MRI is subdivided into overlapping sections, and an optimal affine transformation is calculated between individual MRI sections and the CT. A global deformation vector field is computed based on these transformations. Lastly, the deformation vector field is applied to the MRI to generate a corrected MRI. Further algorithm specifics can be found in a Brainlab technical note.^20^ After running the CDC software, the unmodified CT, corrected MRI, and co‐registration DICOM files were exported to MIM. Finally, the physician re‐contoured targets on both corrected and uncorrected MRIs while blinded to the original contour.

Data analysis of contours was broken into three parts: 1) cross‐comparison of target contours, 2) target dosimetric analysis, and 3) whole brain—target dosimetric analysis. Data analysis began with a comparison of the re‐contoured targets (PTV_RECON_), or corrected targets (PTV_CORR_) and the original planning targets (PTV_ORIG_). All comparisons will be expressed using the following notation: $\Delta TARGET_{SOURCE}^{METRIC}$, where “*TARGET*” represents either PTV or NT (normal tissue), “SOURCE” indicates either re‐contour (RECON) or corrected (CORR), and metric is either volume, minimum dose (*D*_min_), dose to 95% of the PTV (D95%), or volume receiving a given dose (*V*_dose_) (e.g., $\Delta PTV_{RECON}^{volume}$). When referencing a given metric for both sources, the “SOURCE” will be listed as “RECON, CORR” (e.g., $\Delta PTV_{RECON,CORR}^{volume}$). It is implied that Δ for a given source represents the normalized difference relative to the corresponding PTV_ORIG_ statistic.

Cross comparison of targets using similarity coefficients will be presented as: $METRIC_{SOURCE}$, where “METRIC” is either DICE or JACCARD, and “SOURCE” is either RECON, CORR, or “RECON, CORR” when referring to both. Likewise, to ΔTARGET, these RECON and CORR similarity metrics are all calculated relative to the original PTVs.

Volumes of PTVs were quantified by counting the number of voxels in each contour. Changes in number of voxels for individual PTV_RECON_ and PTV_CORR_ were calculated as the percentage change in number of voxels compared to PTV_ORIG_, which was normalized to the voxels in PTV_ORIG_. In addition to calculating changes in contour size, degree of overlap between contours was quantified by calculating DICE and JACCARD similarity coefficients between the new PTV_RECON_ and PTV_CORR_ contours, and the original PTV_ORIG_.^21^, ^22^ Finally, to evaluate the potential benefit of adding uniform margins to the PTV_ORIG_ contours to account for distortion, a 1 mm margin was introduced and the percentage of PTV_CORR_ and NT covered by the expanded PTV_ORIG_ were calculated as a normalized ratio of PTV_ORIG_.

To assess dosimetric plan quality for the various targets, minimum target dose and dose covering 95% of the individual targets was recorded. Changes in target doses for PTV_RECON_ and PTV_CORR_ were calculated as a normalized percent change from PTV_ORIG_.

To investigate changes in normal tissue (NT) dose, the dose delivered to the whole brain, excluding the PTV structures, was evaluated. The whole brain was contoured on the CT in MIM, and target contours were subtracted to create three NT structures, one for each set of targets (ORIG, RECON, CORR). The volume of the NT structures receiving 12 Gy (*V*_12Gy_), 10 Gy (*V*_10Gy_), and 5 Gy (*V*_5Gy_), were recorded. Changes NT dose metrics for NT_RECON_ and NT_CORR_ were calculated as the percentage change from NT_ORIG_, normalized to metrics in NT_ORIG_.

For each calculation, two sets of changes or metrics were generated for the PTV_RECON_ and PTV_CORR_ targets. To discern whether changes in these targets, as compared to PTV_ORIG_, were primarily attributed to variability in contouring or MRI distortion, two‐tailed Student's *t*‐tests were performed between corresponding sets of data.^23^

## 3 RESULTS

### 3.1 Volume changes and similarity comparisons

**FIGURE 2:**
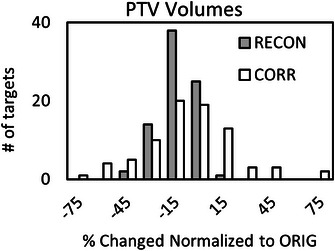
Normalized change in PTV volumes resulting from re‐contouring (RECON) and distortion corrections (CORR), with respect to the original (ORIG).

Total PTV_CORR_ volumes were 1.48% ± 32.92% larger, on average, than PTV_ORIG_ volumes, but there was a large standard deviation across changes in individual PTV_CORR_ volumes. In contrast, recontoured PTV_RECON_ volumes were 6.19% ± 10.95% smaller than PTV_ORIG_ volumes, indicating that recontouring resulted in smaller volumes, on average, compared to contouring in the original planning software. Therefore, similarity in sizes between the PTV_CORR_ and PTV_ORIG_ volumes suggests that enhancement regions on the corrected MRIs increased relative to uncorrected MRIs to account for smaller contour sizes in MIM. Table 1 summarizes average and standard deviations of the ΔPTVs and similarity metrics, along with the *p*‐values from the Student's *t*‐test. Figure 2 presents a histogram comparison of $\Delta PTV_{RECON}^{volume}$ and $\Delta PTV_{CORR}^{volume}$.

**TABLE 1:** Normalized change in PTV volumes, minimum dose to PTV (Δ*D*_min_), and dose to 95% of the PTV (ΔD95%) resulting from re‐contouring (RECON) and after distortion corrections (CORR), with respect to the original (ORIG) volumes.

	RECON and CORR PTV volumes and dose relative to ORIG									
	Δ PTV volume		DICE		JACCARD		Δ *D*_min_		Δ D95%	
	Mean	SD	Mean	SD	Mean	SD	Mean	SD	Mean	SD
RECON	−6.18	10.95	0.89	0.08	0.82	0.12	−0.88	8.77	−0.34	5.88
CORR	1.48	32.92	0.73	0.16	0.60	0.18	−12.90	17.29	−8.68	13.21
*p*‐values	*p* = 0.04		*p* < 0.01		*p* < 0.01		*p* < 0.01		*p* < 0.01	

*Note*: *p*‐values comparing RECON and CORR changes calculated using Student's *t*‐test.

**FIGURE 3:**
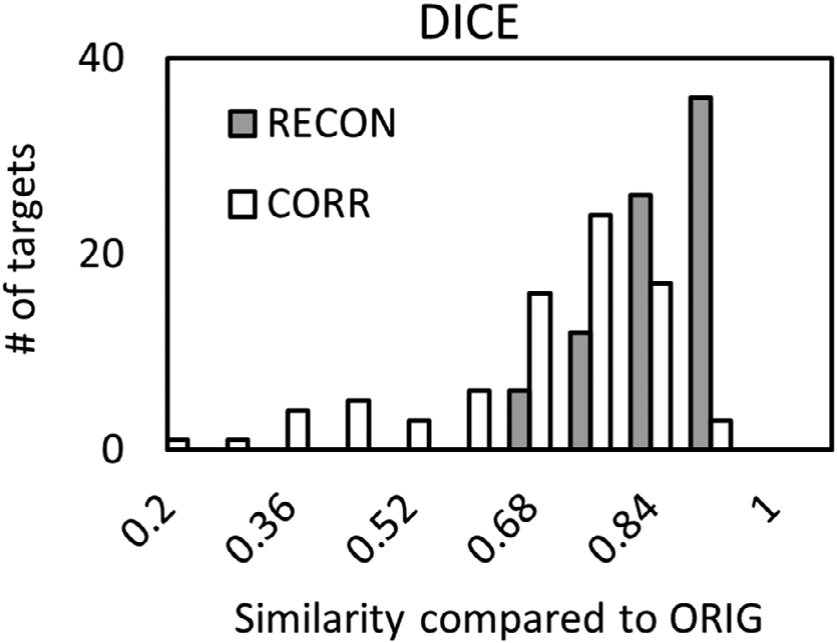
Histograms of DICE similarity coefficients between re‐contoured PTVs (RECON) and distortion‐corrected PTVs (CORR) compared to the original (ORIG) PTVs.

**FIGURE 4:**
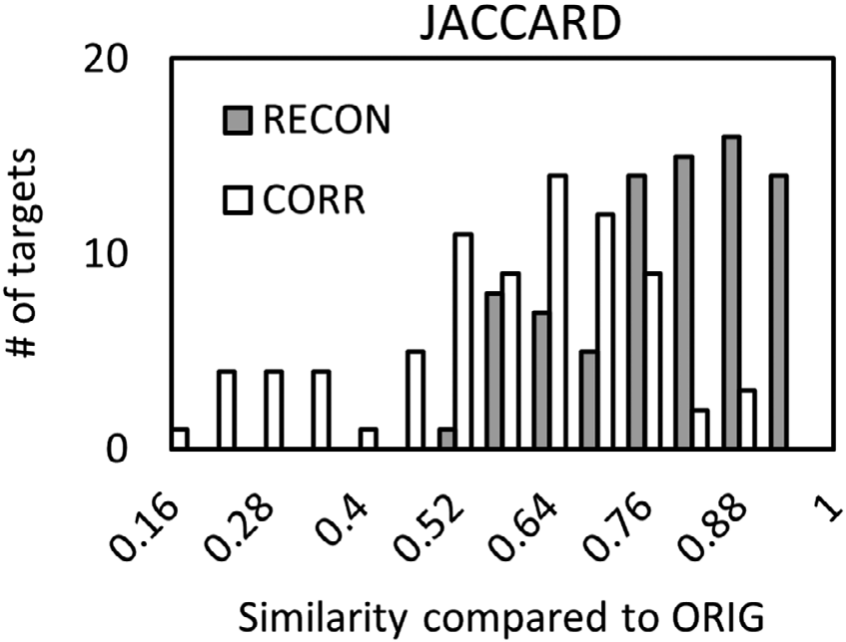
Histograms of JACCARD similarity coefficients between re‐contoured PTVs (RECON) and distortion‐corrected PTVs (CORR) compared to original (ORIG) PTVs.

Although PTV_CORR_ targets had similar average sizes as PTV_ORIG_, there was a significant reduction in similarity metrics compared to PTV_RECON_ (*p* < 0.01). This is consistent with the large standard deviation in $\Delta PTV_{CORR}^{volume}$, indicating that MRI distortion can cause substantial changes in individual target volumes. Moreover, the large standard deviation in $\Delta PTV_{CORR}^{volume}$ with an average close to zero suggests that targets both increased and decreased in size due to distortion. Figures 3 and 4 show histograms of *DICE_RECON, CORR_* and *JACCARD_RECON, CORR_* similarity metrics, respectively.

### 3.2 Dosimetric impacts of distortion

**FIGURE 5:**
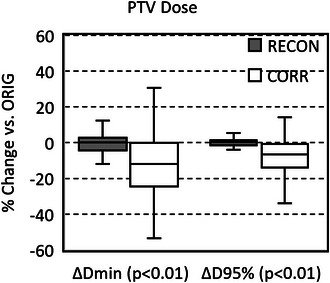
Box‐and‐whisker plots of normalized changes in minimum PTV dose (Δ*D*_min_) and dose to 95% of the PTV (ΔD95%) resulting from re‐contouring (RECON) and after distortion corrections (CORR), with respect to original (ORIG) PTVs. The x‐axis labels show *p*‐values from the Student's *t*‐test comparing RECON and CORR data.

Average reduction in $\Delta PTV_{CORR}^{Dmin}$ (−12.9 ± 17.3%) was significantly lower than reduction in $\Delta PTV_{RECON}^{Dmin}$(−0.88% ± 8.77%) due to intra‐contouring variability (*p* < 0.01). Additionally, average reduction in $\Delta PTV_{CORR}^{D95\%}$ (−8.68% ± 13.21%) was significantly lower than reduction in $\Delta PTV_{RECON}^{D95\%}$ (−0.34% ± 5.89), with a *p* < 0.01. Figure 5 displays box plots of $\Delta PTV_{RECON,\;CORR}^{Dmin}$ and $\Delta PTV_{RECON,CORR}^{D95\%}$. Notably, out of 88 PTV_CORR_ targets, 14 still exhibit more than 5% of their volume that remains uncovered by PTV_ORIG_, even with the inclusion of a 1 mm expansion. In contrast, all PTV_RECON_ targets had 98.5% or more of their volumes covered by PTV_ORIG_ targets with a 1 mm expansion.

**FIGURE 6:**
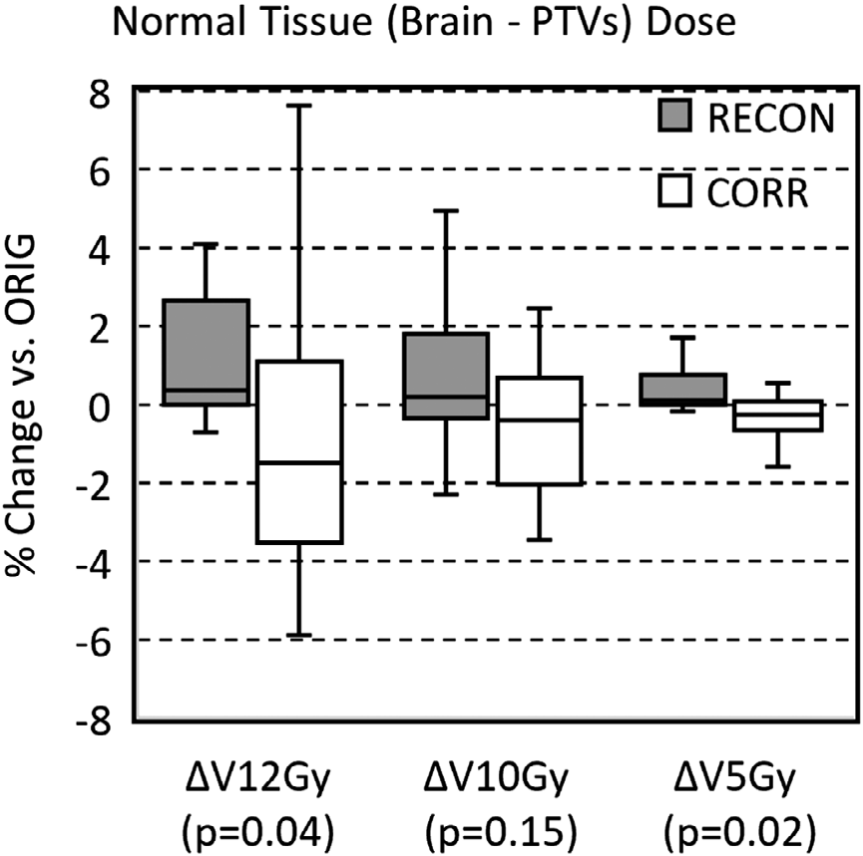
Box‐and‐whisker plots of normalized changes in the volume of normal tissue (brain—PTVs) receiving 12 Gy (ΔV12Gy), 10 Gy (ΔV10Gy), and 5 Gy (ΔV5Gy) resulting from re‐contouring (RECON) and after distortion corrections (CORR), with respect to the original (ORIG) PTVs. The x‐axis labels show *p*‐values from the Student's *t‐*test comparing RECON and CORR data.

Table 2 reports average and standard deviation *ΔNTs*, as well as *p*‐values from the Student's *t*‐test. Figure 6 shows box plots of $\Delta NT_{RECON,\;CORR}^{V12Gy}$, $\Delta NT_{RECON,CORR}^{V10Gy}$, and $\Delta NT_{RECON,CORR}^{V5Gy}$.

**TABLE 2:** Normalized changes in the volume of normal tissue (brain‐PTVs) receiving 12 Gy (ΔV12Gy), 10 Gy (ΔV10Gy), and 5 Gy (ΔV5Gy) dose due to re‐contouring (RECON) and after distortion corrections (CORR), with respect to original (ORIG) normal tissue volumes.

	Normal tissue (brain—PTVs) dose relative to ORIG					
	ΔV12Gy		ΔV10Gy		ΔV5Gy	
	Mean	SD	Mean	SD	Mean	SD
RECON	−0.09	7.39	−0.38	5.67	−0.08	2.04
CORR	−2.14	7.34	−1.42	5.45	−0.61	1.93
*p*‐values	*p* = 0.04		*p* = 0.15		*p* = 0.02	

*Note*: *p*‐values comparing RECON and CORR changes calculated using Student's *t*‐test.

## 4 DISCUSSION

The CDC software was used to correct distortions in frame‐based MRIs, and it was observed that errors encountered were significantly larger than intra‐contouring variability. However, current clinical practice relies on historical data that demonstrates favorable outcomes without such corrections.^24^, ^25^, ^26^ In their investigation, Amaral et al. discovered that distortions exceeding 1 mm were present in 17.5% of cranial tumor studies potentially leading to higher incidence of local recurrence. Nonetheless, the entire cohort exhibited overall good rates of local control.^27^ Consequently, addressing how the CDC algorithm can be effectively integrated into clinical workflows to enhance normal tissue sparing, decrease geometric misses, while preserving favorable overall outcomes will be explored in three sections. First, the characteristics of the distortions found in this study and their impact on clinical plans will be explored. Second, potential applications of the CDC software in a clinical setting will be proposed. Finally, the limitations of this study and potential avenues for future investigation will be discussed.

### 4.1 Impacts of distortion

To assess magnitudes of distortion relative to contouring variability within a single observer, recontouring was employed as an internal control and baseline. It is important to note that the contouring observer was blinded to initial contours, hence some changes in tumor volume were expected. Although the PTV_RECON_ were smaller than the PTV_ORIG_ targets, $\Delta PTV_{RECON}^{volume}$ and similarity coefficients were in line with interobserver variabilities previously reported in the literature for cranial SRS contouring.^28^, ^29^, ^30^, ^31^ The average reduction in size of the PTV_RECON_ compared to the PTV_ORIG_ targets indicates that residual distortion in the original uncorrected MRIs caused an underestimation of the contrast‐enhanced regions in patients. On average, PTV_CORR_ increased and PTV_RECON_ decreased in size compared PTV_ORIG_, however volumetric changes occurred in both directions as shown in Figure 2. Moreover, higher degrees of agreement observed between original and recontoured targets through JACCARD and DICE metrics support contour changes based on distortion having a more pronounced impact on target delineation compared to contouring variability.

For both PTV_RECON_ and PTV_CORR_ individual targets that showed an increase in volume, there was either no change or a reduction in *D*_min_. This can be attributed to increased volumes causing the exterior of the PTVs to extend further into the dose falloff region, resulting in overlap with a lower isodose line. Instances where the target volume increased, without any change in *D*_min_, were likely caused by either shifting of a target without a significant change in conformity or limitations in the significant figures and dose resolution of the software.

While applications of the CDC software led to overall average increases in target size, some targets did decrease in size. These decreasing volumes did not always exhibit the trend of increasing the minimum target dose. In fact, there were instances where target size decreased and the minimum dose either increased, decreased, or remained unchanged. This was because distortion corrections did not always result in a simple uniform contraction of target volumes. Instead, reductions in size were sometimes accompanied by a shift of the target centroid as shown in Figure 1. Such a shift could cause targets to extend further into the dose falloff region, like what was observed with increasing target sizes. Overall, the study found that both increasing and decreasing target volumes associated with CDC corrections could result in lower minimum PTV doses, but only a decrease in target volume could potentially lead to an increase in minimum PTV dose.

The impact of distortion on normal tissue would vary on a case‐by‐case basis in terms of clinical relevance; however, bidirectional changes in PTV dose were accompanied by corresponding bidirectional changes in dose to normal brain tissue. On average across all patients, volumetrics used to evaluate normal tissue (*V*_12Gy_, *V*_10Gy_, *V*_5Gy_) decreased after distortion correction. The $\Delta NT_{\;CORR}^{V12Gy,V5Gy}$ were significantly different (*p* < 0.05) than $\Delta NT_{\;RECON}^{V12Gy,V5Gy}$, while no statistically significant difference was observed for *V*_10Gy_. As with standard deviations between changes in PTV contours, there was a large standard deviation in changes observed in normal tissues. This underscores the difficulty in predicting distortion effects on a cohort of MRI data, even when scanners and inter‐patient geometry stay relatively constant between scans.

While this study primarily focused on dosimetric evaluations of distortion within a clinical patient population, other investigations have examined this phenomenon using phantoms or MRIs with artificially induced distortions. In a recent study, Ohira et al. explored the impact of MR distortion through phantom studies involving single‐isocenter multi‐target treatments.^32^ They observed that the dosimetric deviations due to distortion were less than 5% of the planned value when the target was within 50 mm of the MR image center but increased as the targets extended away from the image center. Similarly, Pappas et al. noted comparable trends of distortion increasing away from the center in their phantom studies. For smaller targets with diameters less than 2 mm, 1 mm distortions caused 5% changes in dose and as target sizes grew to diameters of 20 mm, a 1.5 mm distortion would produce a similar dosimetric effect.^14^ Calvo‐Ortega et al. introduced artificial distortions into MRIs of vestibular schwannoma patients and utilized the CDC algorithm to correct these distortions.^33^ Their findings indicated that the algorithm could correct patients within 0.1 ± 0.1 Gy of the undistorted targets. These studies collectively underscore the potential impact of distortion in cranial stereotactic radiosurgery treatments.

### 4.2 Clinical implementation of the CDC algorithm

The availability of quantifying and correcting patient‐specific MR distortion introduces a possible paradigm shift in target contouring and use of margins for radiosurgery. When considering data on distortion in conjunction with acceptable clinical outcomes, radiosurgeons have several viable courses of action. The first option is to continue common clinical practices of not adding a margin to PTVs for some frame‐based systems, while loosely conforming prescription isodose lines to targets to ensure 100% coverage. The second option is to introduce a planning margin during PTV generation to account for planning and treatment uncertainties, including distortion, while more tightly conforming dose to the contour. However, previous investigations have shown that magnitudes of distortion and the effect on each target is non‐uniform, rendering a uniform distortion‐correction margin inappropriate.^14^, ^15^, ^34^ Despite the implementation of a 1 mm margin in this study, some tumors still exhibited distortion beyond this expansion, indicating this approach may mitigate some, but not all distortion‐related risks. Consequently, adding additional planning margins alone would not eliminate the potential for a geometric miss caused by distortion, while increasing the risk of radionecrosis by exponentially enlarging treated tissue volumes. An alternative approach would be to generate a composite target that includes both initial targets without distortion correction and distortion‐corrected targets, resulting in a likely lenticular volume of normal tissue included in treatment volumes. This approach may potentially avoid the aforementioned exponential treatment volume increase. Lastly, a target may be treated solely based on algorithmic estimation and correction of distortion, with or without the inclusion of a planning margin and its associated risks and benefits. There may be instances where a combination of these approaches is considered, rather than uniformly implementing a single approach based on target size, location, and physician comfort when presented with patient‐specific distortion correction calculations.

While the CDC software has been investigated for correcting patient‐specific distortion, system‐level MR distortion may be present in treatment planning MRIs. These system‐level distortions can stem from machine‐related issues, such as inadequate magnetic shimming and eddy current compensation.^17^, ^35^ Moreover, significant distortion errors may occur if correction options in the scanning protocol are unintentionally disabled, resulting in geometric errors up to 5.0 mm that may go unnoticed by certain observers.^36^ System‐level distortions are more likely to occur when an SRS program utilizes MRIs from external radiology departments for planning, as scanner quality assurance in such cases tends to prioritize diagnostic contrast rather than sub‐millimeter geometric accuracy. Additionally, system‐level distortions generally exhibit larger magnitudes than patient distortions, thereby increasing the likelihood of substantial geometric errors.^5^ In such instances, the utilization of the CDC software would offer the advantage of allowing a practice to use images from different MRI scanners, while preserving high‐level spatial accuracy required for SRS deliveries.

### 4.3 Future directions and study limitations

While CTs can be acquired for planning and stereotactic frame definition, current research is exploring generation of synthetic CTs from planning MRIs, which would eliminate the requirement for a separate CT scan for treatments.^37^ However, one limitation of this technique is that CTs would inherit the geometric distortion present in source MRIs. To mitigate this effect, the CDC software can be employed to correct planning MRs using a recent diagnostic cranial CT, thereby reducing geometric uncertainty within the cranial region of synthetic CTs.

Although the CDC algorithm shows promise in improving geometric accuracy, like any distortion correction algorithm, it poses challenges when it comes to in vivo validation. Retif et al. examined the precision of the CDC algorithm in rectifying distortions in a cranial phantom and found the algorithm corrected 96% of voxels to within 0.5 mm.^16^ However, the CDC algorithm is designed to use anatomy contained with the patient's CT for its correction, therefore validation in anthropomorphic phantoms may not sufficiently account for the full range of CT and MRI densities and settings within the clinical framework. Like other clinical anatomical algorithms, the acceptance of a solution requires close evaluation on a case‐by‐case basis. This holds true for all imaging fusions, whether they involve distortion, deformation corrections, or fixed‐based fusion solutions.

While the inability to validate the distortion corrections in situ represents a limitation of this study, another constraint lies in the incapacity to differentiate between distortion and registration errors between the planning CT and MR images. Although the co‐registration of the images was confirmed by a neurosurgeon, radiation oncologist, and physicist during the planning process, distortion in the initial MRIs could introduce unavoidable registration errors when attempting to co‐register targets throughout the brain, especially where geometric spacing was inconsistent between the CT and MR images. The CDC algorithm's initial step of subdividing the brain into smaller sections before deformable registration would help mitigate the effects of these global registration errors. From a therapeutic perspective, these registration errors would impact treatment whenever the CT was used for patient alignment. This CT could be employed to define the stereotactic frame, or the CT anatomy of the skull could be used to validate patient positioning through co‐registration to a cone‐beam CT acquired immediately before dose delivery on the treatment machine.

Another constraint in this study was the restricted scope of analysis, which focused solely on brain metastasis from a single institution. Various factors, including target size and location, magnetic field strength, acquisition parameters, and MR scanner‐specific distortion correction algorithms, could influence distortion magnitudes. Furthermore, the added complexities of physician variability in contouring MR‐enhancing regions and institutional variations in required dose conformality further impact distortion effects. Consequently, further validation of the CDC software across multiple institutions for various treatment indications is imperative to enhance the global significance of the findings. In addition to its application across multiple institutions to quantify overall impact of MRI distortions, the CDC software can also improve dosimetric reporting in clinical trials, where data errors can impede meaningful conclusions during meta‐analysis. Therefore, integrating the CDC software into the planning process presents an opportunity to adopt a more robust approach that minimizes distortion errors arising from both machines and patients of a large cohort of centers participating in clinical trials.

## 5 CONCLUSION

This study retrospectively applied the CDC software to correct distortion in 18 MRIs used for target delineation in frame‐based SRS plans. The objective was to evaluate the effect of distortion‐induced changes in target shapes, target dose, and normal tissue dose. The results revealed that the distortion in MRIs used for SRS caused significant changes in most metrics used for evaluating quality and efficacy of SRS plans. These findings suggest that accounting for MRI distortions is critical to ensure accurate SRS treatment planning and delivery. To reduce residual distortion in treatment planning MRIs, which may persist even after scanner‐specific corrections, additional patient‐specific correction methods such as the CDC software can be implemented.

## AUTHOR CONTRIBUTION

All authors have made significant contributions to the research and writing of this article. They have all reviewed this manuscript and have approved it for submission to this journal.

## CONFLICT OF INTEREST STATEMENT

The authors declare no conflicts of interest.
